# ALCAM shedding at the invasive front of the tumor is a marker of myometrial infiltration and promotes invasion in endometrioid endometrial cancer

**DOI:** 10.18632/oncotarget.24625

**Published:** 2018-03-30

**Authors:** Laura Devis, Elena Martinez-Garcia, Cristian P. Moiola, Maria Teresa Quiles, Maria Antonia Arbos, Tomita Vasilica Stirbat, Françoise Brochard-Wyart, Ángel García, Lorena Alonso-Alconada, Miguel Abal, Berta Diaz-Feijoo, William Thomas, Sylvie Dufour, Gemma Mancebo, Francesc Alameda, Jaume Reventos, Antonio Gil-Moreno, Eva Colas

**Affiliations:** ^1^ Biomedical Research Group in Gynecology, Vall Hebron Research Institute, Universitat Autònoma de Barcelona, CIBERONC, Barcelona, Spain; ^2^ Grup de Recerca en Cirugia General, Institut de Recerca Vall d'Hebron, Hospital Universitari Vall d'Hebron, Universitat Autònoma de Barcelona, Barcelona, Spain; ^3^ Institut Curie, CNRS, Paris, France; ^4^ Pathology Department, Vall Hebron University Hospital, Barcelona, Spain; ^5^ Translational Laboratory, Medical Oncology Department, Complexo Hospitalario Universitario de Santiago, SERGAS, CIBERONC, Santiago de Compostela, Spain; ^6^ Gynecological Oncology Department, Vall Hebron University Hospital, Barcelona, Spain; ^7^ Department of Natural Sciences, Colby-Sawyer College, New London, New Hampshire, United States of America; ^8^ Institut Curie, CNRS, Paris, France; ^9^ Present address: Université Paris Est, Faculté de Médecine, Créteil, France, INSERM, U955, Equipe 6, Créteil, France; ^10^ Gynecological Oncology Department, Hospital del Mar, PSMAR, Barcelona, Spain; ^11^ Pathology Department, Hospital del Mar, Barcelona, Spain; ^12^ Basic Sciences Department, International University of Catalonia, CIBERONC, Barcelona, Spain

**Keywords:** ALCAM, endometrial cancer, myometrial invasion, MMP-9, ETV5

## Abstract

Endometrial cancer (EC) is the sixth deadliest cancer in women. The depth of myometrial invasion is one of the most important prognostic factors, being directly associated with tumor recurrence and mortality. In this study, ALCAM, a previously described marker of EC recurrence, was studied by immunohistochemistry at the superficial and the invasive tumor areas from 116 EC patients with different degree of myometrial invasion and related to a set of relevant epithelial and mesenchymal markers. ALCAM expression presented a heterogeneous functionality depending on its localization, it correlated with epithelial markers (E-cadherin/β-catenin) at the superficial area, and with mesenchymal markers at the invasive front (COX-2, SNAIL, ETV5, and MMP-9). At the invasive front, ALCAM-negativity was an independent marker of myometrial invasion. This negativity, together with an increase of soluble ALCAM in uterine aspirates from patients with an invasive EC, and its positive correlation with MMP-9 levels, suggested that ALCAM shedding by MMP-9 occurs at the invasive front. *In vivo* and *in vitro* models of invasive EC were generated by ETV5-overexpression. In those, we demonstrated that ALCAM shedding was related to a more invasive pattern and that full-ALCAM recovery reverted most of the ETV5-cells mesenchymal abilities, partially through a p-ERK dependent-manner.

## INTRODUCTION

Endometrial cancer (EC) is the most common gynecologic cancer and the sixth most deathly cancer in women from western countries [1]. Myometrial invasion is one of the most important prognostic factors of EC and represents an increase in the rate of recurrence and a decrease in the 5-year survival [2]. The first step of myometrial invasion is characterized by the dissociation of tumor cells from the epithelial layer of the endometrial glands, and their subsequent infiltration through the basement membrane into the adjacent layer, the myometrium [3]. For that to happen, several authors suggest that epithelial tumor cells need to undergo an epithelial-to-mesenchymal transition (EMT). Moreover, it is known that in invading tumors this process takes place at the invasive front [4–6]. EMT is a dynamic process controlled by external signals coming from the tumor cells microenvironment, that involves cellular loss of polarity, loosen cell-cell contacts, reorganization of cytoskeleton and acquisition of plasticity, motility and invasive capabilities. Multiple factors have been described as being responsible for producing EMT in EC, such as ETV5, estrogen and progesterone receptors, and TGF-β, among others [3].

Activated-leukocyte cell adhesion molecule (ALCAM) is a cell-cell adhesion molecule of 105-kDa from the immunoglobulin superfamily (IgSF) that participates either in homotypic (ALCAM-ALCAM) or heterotypic (ALCAM-CD6) interactions between adjacent cells [7]. ALCAM presents 5 extracellular immunoglobulin domains (D1–D5), comprising 2 NH2-terminal, membrane-distal variable-(V)-type and 3 membrane-proximal constant-(C2)-type Ig folds, a transmembrane-region and a short carboxy-terminal cytoplasmic tail. The extracellular domains provide two structural and functional modules, responsible for ligand affinity (D1-D2) that mediates receptor *trans-trans*-interactions between opposing cells, and avidity (D3-D5). The strength of the avidity is controlled by recruitment of ALCAM molecules to the site of cell-cell contact and by receptor *cis*-oligomerization via the C-type immunoglobulin domains. Coordination of both modules is necessary for stable ALCAM-ALCAM interaction, leading to the formation of a tight bilayered ALCAM network [8]. Both, homotypic and heterotypic interactions are mediated through its amino-terminal V domain (D1). ALCAM-ALCAM interactions are described as a D1–D1 interaction, while in ALCAM–CD6 interactions the ALCAM D1 domain binds to the CD6 membrane-proximal scavenger receptor cysteine rich (SRCR) domain [9, 10].

Activation of ALCAM-mediated adhesion is dynamically regulated through actin cytoskeleton-dependent clustering. Zimmerman et al. described the interaction between ALCAM and the actin cytoskeleton [11]. This interaction could be allowed by the presence in its short cytoplasmic tail of a positive-charge-rich domain (PCRD) as well as a PDZ-binding motif KTEA placed at the C-terminus [12]. The inhibition of actin polymerization by low levels of cytochalasin D stimulates homotypic ALCAM–ALCAM interactions, increases protein lateral mobility, and the formation of ALCAM clusters in the cell surface, demonstrating that cytoskeleton regulates ALCAM-mediated adhesion [13]. Moreover, ALCAM has been observed to form a supra-molecular complex with Syntenin-1 and Ezrin [14]. PKCα also plays a role in the cytoskeleton-dependent avidity modulation of ALCAM and regulates the supra-molecular complex formed by ALCAM-Ezrin [15].

ALCAM expression in the cell surface can be regulated by endocytosis or protein cleavage. In fact, ALCAM co-localizes with clathrin in a process that is regulated by ubiquitination and dependent on ERK phosphorylation [16, 17]. ALCAM levels can be also regulated by protein shedding via ADAM-17 metalloproteinase [18], generating a soluble 96-kDa form and a truncated membrane-bound. ALCAM is widely distributed within tissues and its function has been reported in extensive biological processes [19]. In the last years, it has been associated to the tumorigenesis of many cancers [20–27]. However, ALCAM expression and localization within the tumor cell have generated great controversy, and its relation to prognosis remain still unclear [28]. In addition, ALCAM shedding has been reported in the serum of breast, ovarian, thyroid and pancreatic cancer patients and in all the cases, related to poor clinical outcome [24, 29–31]. We have previously demonstrated that ALCAM is an important player in endometrioid endometrial cancer (EEC) dissemination [32]. In fact, we described that ALCAM-positivity is a marker of recurrence in early stage EEC and promotes migration and invasion *in vitro* and *in vivo*.

In this study, we go further in our understanding of ALCAM in EC tumorigenesis by evaluating its full-form expression in two different areas of the tumor and its relation with key molecules associated to the epithelial and mesenchymal context. Immunohistochemical staining for ALCAM in tissues was conducted with an antibody directed against the extracellular domain of the protein. Since, ALCAM can be proteolytically processed by metalloproteinases, thereby generating a soluble ALCAM component and a truncated membrane-bound ALCAM containing the transmembrane and cytoplasmic domain, the antibody recognized the non-cleaved protein.

We found a switch in the functional mode of ALCAM in EEC. Whereas at the superficial area of the tumor ALCAM correlated with the E-Cadherin/β-Catenin adhesion complex, at the invasive front ALCAM could be shed by MMP-9 in poor prognosis scenarios. Moreover, we evaluated the shed ALCAM, or soluble form (sALCAM), in uterine aspirates as a potential diagnostic tool to predict myometrial invasion.

## RESULTS

### ALCAM expression presented a different correlation profile depending on its localization within the tumor

We studied the immunohistochemical expression of ALCAM extracellular domain (non-cleaved protein) and a representative set of epithelial (E-cadherin/β-catenin major adhesion complex) and mesenchymal molecules involved in EEC dissemination (ETV5, COX-2, SNAIL, SLUG, MMP-2 and -9), in both the superficial and the invasive areas of the tumor. As our previous results indicated a different behavior of ALCAM regarding tumor differentiation, analyses were performed in the entire population, and in the well-differentiated (GI) and poorly-differentiated (GII-III) subcohorts [32].

**Figure 1:**
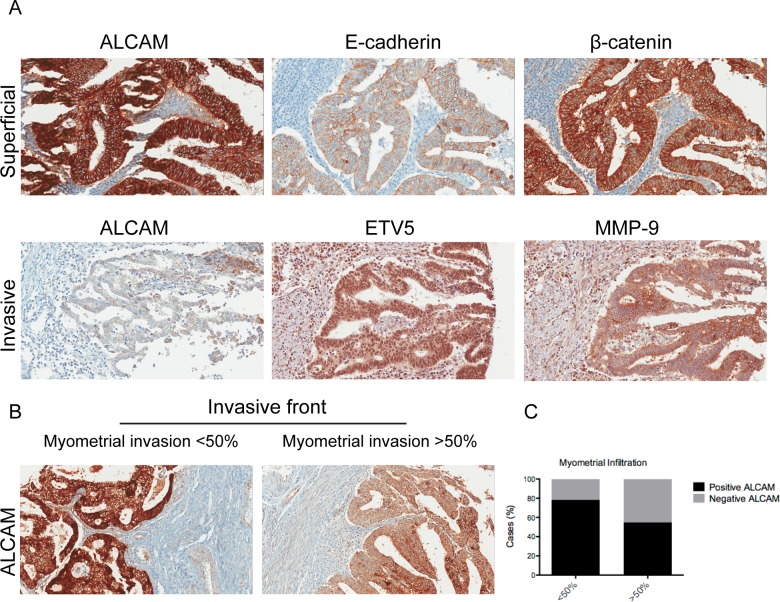
ALCAM expression presented a different correlation profile depending on its localization within the tumor and its decrease at the invasive front is a marker of myometrial invasion **(A)** Upper: Full-length ALCAM was significantly and positively correlated with the E-cadherin/β-catenin complex at the superficial tumor and is mainly localized at the cell membrane. Down: At the invasive front, full-length ALCAM is significantly inversely correlated with MMP-9 and ETV5, in the moderately-poorly differentiated EEC. **(B)** ALCAM-negativity, at the invasive front, is a marker of myometrial invasion **(C)** At the invasive front, ALCAM-negative tumors increased from a 21.8% in patients without myometrial invasion to a 45.1% in patients presenting myometrial infiltration (p-value= 0.014).

Univariate regression analyses were performed as a first approach to evaluate the association between ALCAM and epithelial and mesenchymal markers. Variables that significantly correlated with ALCAM (Supplementary Table 1) were included in multivariate linear regression models. ALCAM presented a significant positive correlation with the major adhesion complex at the superficial area of the tumor. Whilst β-catenin was retained as an outcome variable in all patients and in the well-differentiated cohort (R=0.437 and 0.394, respectively), E-cadherin was retained in the moderately-poorly differentiated tumors (R=0.445) (Supplementary Table 2, Figure 1A). By contrast, at the invasive front of the tumor, SNAIL, COX-2 and MMP-9 were retained as outcome variables of the model, in all patients. COX-2 and SNAIL were positively correlated with ALCAM, whilst MMP-9 was negatively correlated. The best regression adjustment was reached in the moderately-poorly differentiated subcohort of patients, in which the model retained ETV5 and MMP-9 (R=0.559). Both molecules showed a negative correlation with ALCAM. Altogether, the results suggested a different functional profile of ALCAM according to its tumor localization, i.e. whilst it correlated with epithelial markers at the superficial tumor, correlations with mesenchymal markers were observed at the invasive front.

### ALCAM-negativity at the invasive front of the tumor is a marker of myometrial invasion

We further investigated the role of ALCAM at the different tumor areas. At the superficial area, ALCAM expression was unrelated to the clinical parameters of myometrial invasion, tumor grade, and tumor progression. However, it acted as a marker of tumor progression and invasion when analyzed at the invasive front. In this tumor area, ALCAM significantly decreased along tumor stages and in tumors with myometrial invasion >50% (Table 1, Figure 1B-1C). In fact, the potential of ALCAM-negativity as an independent prognostic factor of myometrial invasion was confirmed by multivariate logistic regression analysis with an OR 3.273, together with the tumor grade (OR 3.484) (Table 2).

**Table 1:** ALCAM and clinical parameters

Variable, No. (%)	Σ	ALCAM Positive^*^	ALCAM Negative^†^	P-value
**Superficial area of the tumor**				
**FIGO**	116	82 (70.7)	34 (29.3)	0.208
IA	56	42 (75.0)	14 (25.0)	
IB	41	28 (68.3)	13 (31.7)	
II	15	11 (73.3)	4 (26.7)	
III	4	1 (25.0)	3 (75.0)	
**Grade**	116	82 (70.7)	34 (29.3)	0.131
Grade I	79	60 (75.9)	19 (24.1)	
Grade II	24	13 (54.2)	11 (45.8)	
Grade III	13	9 (69.2)	4 (30.8)	
**Myometrial infiltration**	116	82 (70.7)	34 (29.3)	0.541
< 50%	58	43 (74.1)	15 (25.9)	
≥ 50%	58	39 (67.2)	19 (32.8)	
**Invasive front of the tumor**				
**FIGO**	106	71 (67.0)	35 (33.0)	0.004^**^
IA	53	42 (79.2)	11 (20.8)	
IB	36	21 (58.3)	15 (41.7)	
II	13	8 (61.5)	5 (38.5)	
III	4	0 (0.0)	4 (100.0)	
**Grade**	106	71 (67.0)	35 (33.0)	0.213
Grade I	72	52 (72.2)	20 (27.8)	
Grade II	22	13 (59.1)	9 (40.9)	
Grade III	12	6 (50.0)	6 (50.0)	
**Myometrial infiltration**	106	71 (67.0)	35 (33.0)	0.014^*^
< 50%	55	43 (78.2)	12 (21.8)	
≥ 50%	51	28 (54.9)	23 (45.1)	

^*^Positive ≥ 65 ^†^Negative < 65.

**Table 2:** Multivariate logistic regression model related to the myometrial invasion ≥50% (N = 89)

Variable	OR	95% Interval confidence	P-value
**Grade**			
Differentiated tumors (Grade I)	1		
Moderateley-poorly differentiated tumors (Grade II-III)	3.484	(1.284-9.524)	0.014^*^
**ALCAM**			
Positive	1		
Negative	3.273	(1.200-8.932)	0.021^*^
**MMP-9**			
Positive	1		
Negative	1.629	(0.602-4.409)	0.337
**ETV5**			
Positive	1		
Negative	0.556	(0.194-1.592)	0.274

ALCAM maintained a close and inverse relation with MMP-9 at the invasive front of the tumor (p=0.004; OR 0.26) (Table 3). This relation was only preserved in poor prognosis tumors when the cohort was divided according to clinical parameters, i.e. either in tumors with moderately-poorly differentiated EEC histology (p=0.02; OR 0.08), in patients presenting >50% of myometrial infiltration (p=0.016; OR 0.19), and in ETV5-positive tumors (p=0.009; OR 0.22). Altogether, our results demonstrated that ALCAM-negativity is a marker for myometrial invasion at the invasive front. Its close and inverse relation with MMP-9 in poor prognosis scenarios, led us speculate that ALCAM-negativity might be due to an increased shedding of the protein by the metalloproteinase at the invasive front of poor prognostic tumors.

**Table 3:** ALCAM and MMP-9 correlation at the invasive front of the tumor

Variable, No. (%)	Σ	ALCAM Positive^*^	ALCAM Negative^†^	P-value	OR (Positive/Negative)
**All**	95	64 (67.4)	31 (32.6)	0.004^**^	0.26 (0.10-0.66)
MMP-9 Positive	47	25 (53.2)	22 (46.8)		
MMP-9 Negative	48	39 (81.3)	9 (18.8)		
**Clinical parameters**					
**Grade**					
** Differentiated tumors**	64	46 (71.9)	18 (28.1)	0.162	N.S
MMP-9 Positive	26	16 (61.5)	10 (38.5)		
MMP-9 Negative	38	30 (78.9)	8 (21.1)		
**Moderately- Poorly differentiated tumors**	31	18 (58.1)	13 (41.9)	0.020^*^	0.08 (0.03-0.63)
MMP-9 Positive	21	9 (42.9)	12 (57.1)		
MMP-9 Negative	10	9 (90.0)	1 (10.00)		
**Myometrial infiltration <50%**	53	41 (77.4)	12 (22.6)	0.190	N.S
MMP-9 Positive	25	17 (68.0)	8 (32.0)		
MMP-9 Negative	28	24 (85.7)	4 (14.3)		
**Myometrial infiltration ≥50%**	42	23 (54.8)	19 (45.2)	0.016^*^	0.19 (0.03-0.65)
MMP-9 Positive	22	8 (36.4)	14 (63.6)		
MMP-9 Negative	20	15 (75.0)	5 (25.0)		
**Molecular features**					
**ETV5 Positive**	64	40 (62.5)	24 (37.5)	0.009^**^	0.22 (0.07-0.69)
MMP-9 Positive	37	18 (48.6)	19 (51.4)		
MMP-9 Negative	27	22 (81.5)	5 (18.5)		
**ETV5 Negative**	25	19 (76.0)	6 (24.0)	1.000	N.S
MMP-9 Positive	7	5 (71.4)	2 (28.6)		
MMP-9 Negative	18	14 (77.8)	4 (22.2)		

^*^Positive ≥ 65 ^†^Negative < 65.

### ALCAM shedding takes place in tumors with myometrial invasion and correlates with MMP-9 expression

**Figure 2:**
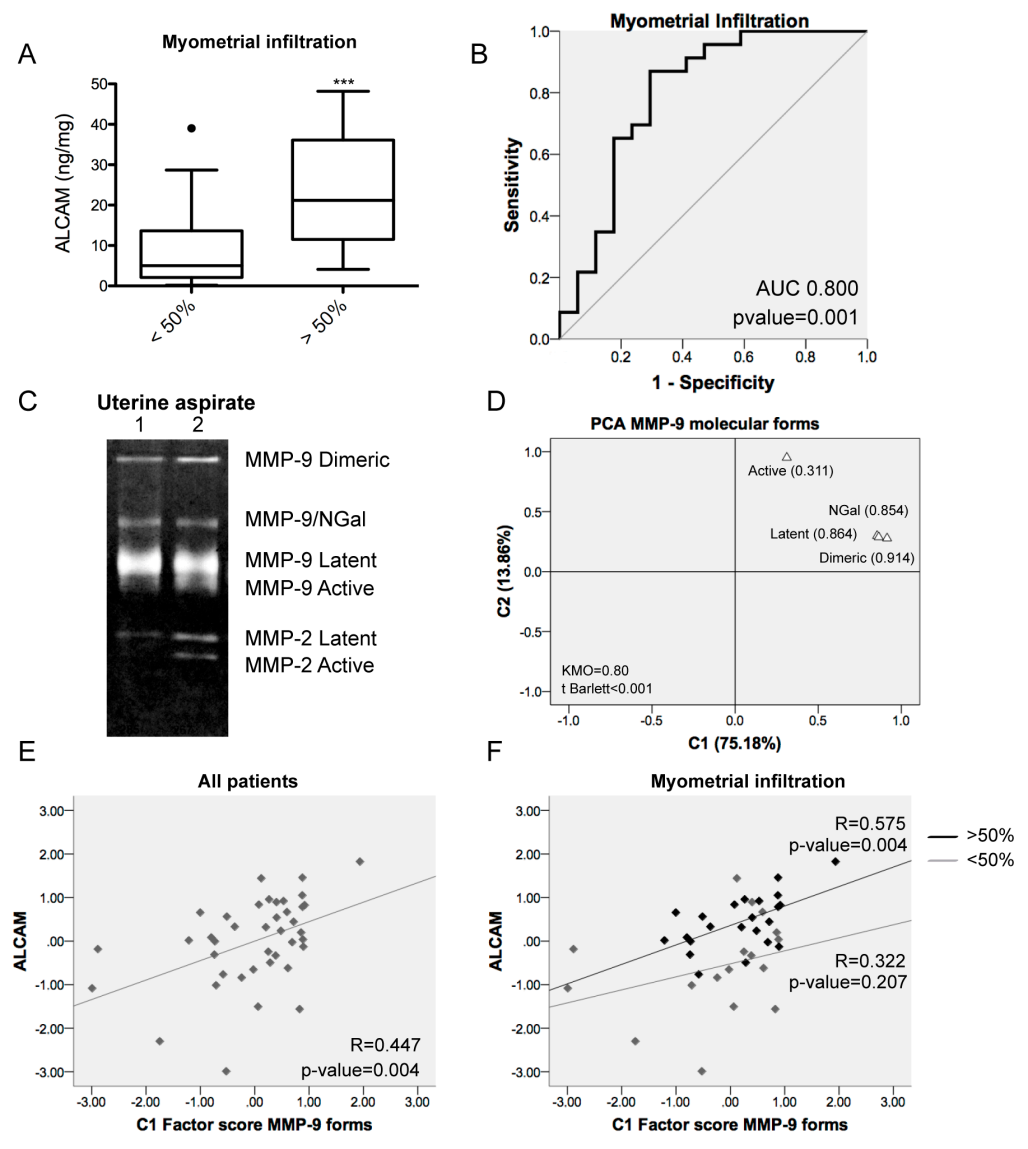
Soluble ALCAM detected in uterine aspirates is a marker of myometrial invasion and correlates with MMP-9 **(A)** Soluble ALCAM detected by ELISA in uterine aspirates was significantly increased in patients presenting myometrial invasion (p-value<0.001). ALCAM values were normalized to total protein amount from the uterine aspirates. **(B)** ROC curve of sALCAM individual marker demonstrated its prognostic value to discriminate patients with myometrial invasion (AUC=0.800; p-value=0.001). **(C)** Two different examples of gelatin zymography from uterine aspirates and detection of the different forms of MMP-9 and MMP-2. **(D)** Principal component analysis from the MMP-9 detected molecular forms evidenced two principal components: C1 (75.18% of the variability) and C2 (13.86% of the variability). The factor loadings, correlation between the original variables and the C1 component, of each form can be observed in the graph. The KMO obtained was 0.80 and the t Barlett <0.001. **(E)** The principal component C1 and the sALCAM were significantly correlated in all patients (R=0.447; p-value=0.004). **(F)** The correlation between the C1 component and sALCAM was significant in patients with myometrial invasion >50% (R=0.575; p-value=0.004).

In order to evaluate the possible association between ALCAM shedding and myometrial invasion, we analyzed the expression of soluble ALCAM (sALCAM) by ELISA, in 40 uterine aspirates from moderately-poorly differentiated EEC patients with different depth of myometrial infiltration. The uterine aspirate is a bodyfluid that is collected from inside of the uterine cavity, and is mainly formed by the secretion of the tumor cells and other cells from the endometrium, enabling the detection of cleaved ALCAM produced by the tumor. We confirmed that sALCAM was significantly increased in patients presenting myometrial invasion >50% (Figure 2A). Moreover, ROC analysis showed that sALCAM in uterine aspirates is a significant predictor of myometrial infiltration (AUC 0.8; p=0.001) (Figure 2B). In fact, when ALCAM is set at a cut-off of 9.375 ng/mg, the model presents a sensitivity of 87% and a specificity of 70.6%.

Then, the relation between sALCAM and MMP-9 was assessed in uterine aspirates by performing a gelatin zymography of the same patients (Figure 2C). Linear regression analyses (Supplementary Table 3) evidenced a significant positive correlation between sALCAM with all MMP-9 detected forms, with the exception of the more labile active form. No correlation was found with the MMP-2 forms (data not shown). In order to avoid the problems derived from the simultaneous use of several measurements that reflect expressions of molecular forms of the same enzyme (Supplementary Table 3), PCA was addressed to explore the close relationship among the four MMP-9 forms and reduce their number before performing regression analysis. Two main components C1, which explains 75.18% of the variability of the original dataset, and C2 which only explains 13.86%, were extracted (Figure 2D). Regression analyses showed a strong and positive correlation between sALCAM and C1, and specifically in patients presenting myometrial invasion >50% (R=0.575; p=0.004) (Figure 2E, 2F). These results suggested that at the invasive front of the tumor, ALCAM extracellular shedding by MMP-9 could be an important process in myometrial invasion.

### ALCAM and MMP-9 are important actors at the invasive front of an *in vivo* model of EEC dissemination

The Hec1A-ETV5 overexpression model has been extensively used to mimic the process of tumor invasion in EEC [33–35], as ETV5 overexpression is known to induce EMT in EEC and has been reported at the invasive front of EEC tumors, promoting migration and invasion *in vitro* and *in vivo*. Furthermore, the parental Hec1A cell line is representative of moderately-poorly differentiated EEC tumor with epithelial-like morphology [33, 36]. To study ALCAM in a controlled environment, which represents a realistic approach towards the process of dissemination of EC, we generated orthotopic murine models with Hec1A (n=5) or its ETV5-overexpressing cells (n=4), directly injected into the uterus of the mice. In this model, tumor cells are localized in the same microenvironment of the original tumor, resembling the processes of tumor growth and myometrial infiltration under endometrial stimuli.

**Figure 3:**
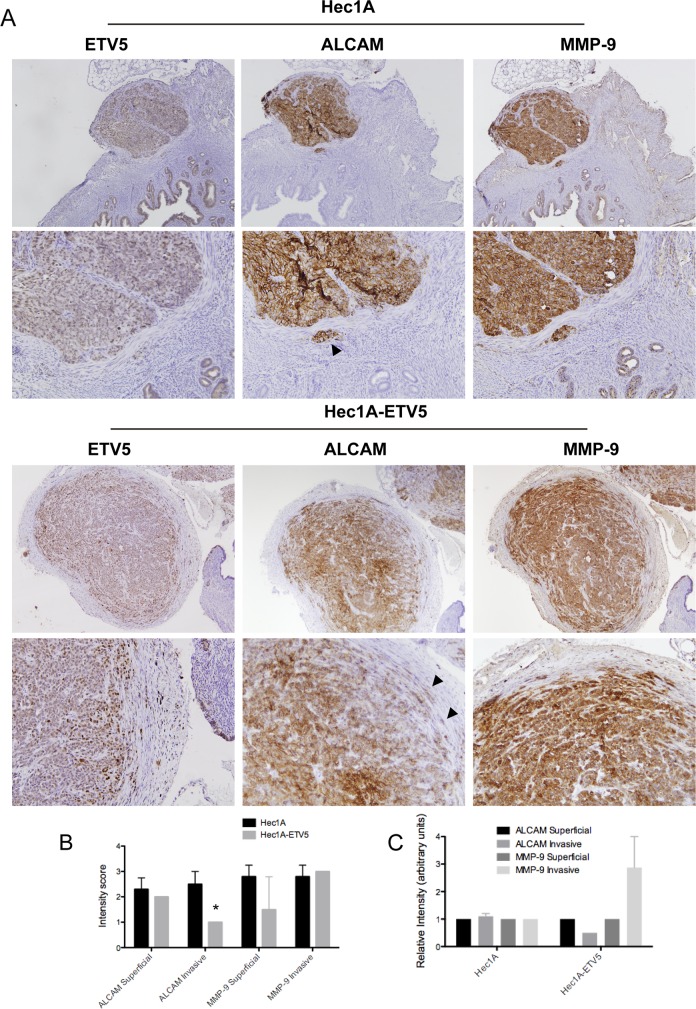
ALCAM is decreased at the invasive front of primary tumors of a controlled model of EEC dissemination **(A)** Upper: Immunohistochemistry of ETV5, ALCAM and MMP-9 in Hec1A control mice. ALCAM and MMP-9 presented a homogeneous staining between the superficial and invasive front or disseminated cells. Black arrow signals a cluster of disseminated cells released from the primary tumor. Down: Immunohistochemistry of ETV5, ALCAM and MMP-9 in Hec1A-ETV5 mice. ALCAM expression was decreased at the invasive front of the tumor, concomitant with an increase of MMP-9 expression. Black arrows evidenced disseminated cells in finger-strand or individual cells, released from the ETV5-overexpressed primary tumor. **(B)** Representation of the intensity of staining of ALCAM and MMP-9. Only, ALCAM expression was significantly decreased at the invasive front of the ETV5-overexpressing mice, compared to control (^*^p<0.05). **(C)** Relative intensity of ALCAM and MMP-9 markers at the invasive front compared to the superficial tumor. While in the control mice, both markers where homogeneous across the section, in the ETV5-overexpressing mice we observed a decreased in ALCAM expression concomitant with an increase in MMP-9 expression in the invasive front of the primary tumors.

As expected, the tumors generated by the ETV5-overexpressing cells presented larger myometrial invasion and tumor burden (Supplementary Figure 1A-1B). While in the ETV5-overexpressing tumors, we observed disseminated cells in finger-like strands or single-cells, projecting into the stroma at the invasive front; in the Hec1A tumors, cells invaded forming clusters (see arrows in Figure 3A), as previously observed by Muinelo-Romay et al. *in vitro* [37]. The immunohistochemical staining of uncleaved ALCAM, MMP-9, and ETV5 in the mice's primary tumors unveiled that ALCAM expression is reduced at the invasive front of ETV5-overexpressing compared to Hec1A tumors (p<0.05; Figure 3B), and moreover, the pattern of expression is modified from a very membranous staining in Hec1A tumors to a diffuse cytoplasmic staining in ETV5-overexpressing cells (Figure 3A).

Interestingly, the intensity of ALCAM and MMP-9 showed no variation between the superficial or the invasive area in Hec1A tumors, but their intensity was altered in the ETV5-overexpressing tumors. In those, MMP-9 increased concomitant with a decrease in ALCAM from the superficial area to the invasive front of the tumor (Figure 3, Supplementary Figure 1C).

As a result of the *in vivo* model, we evidenced that the cell-cell contacts of the Hec1A invading cells seem to be preserved, as shown by the highly collective migration and a homogeneous ALCAM expression. However, the ETV5-overexpressing invading cells were more prone to present switching between thin cords and single-cells, both related to decreased or more transient contacts and higher rate of cleaved ALCAM expression. We finally confirmed that in an invasive scenario, ALCAM and MMP-9 are important actors at the invasive front of the tumor.

### Recovery of ALCAM expression reduced the aggressiveness of invasive EEC cells

**Figure 4:**
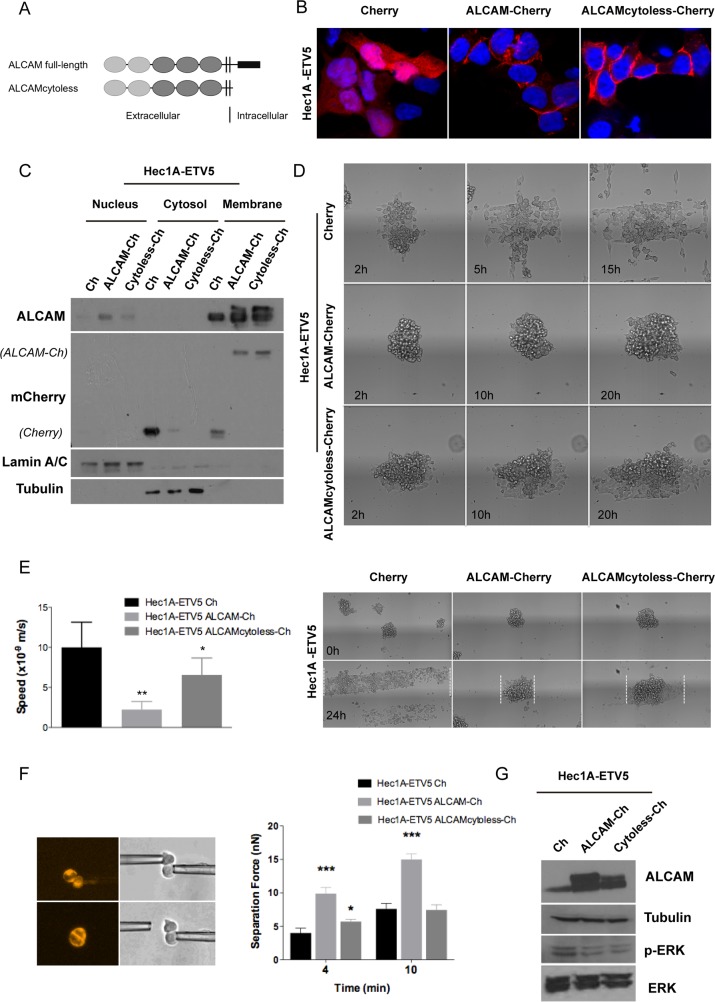
ALCAM overexpression in mesenchymal Hec1A-ETV5 cells decreased cell migration and enhanced cell-cell adhesion **(A)** Representation of the two vectors constructed to overexpress ALCAM: full-length ALCAM and ALCAMcytoless inserted in the pmCherry-N1 construct. **(B)** Images of the Hec1A-ETV5 cell line transfected with the pmCherry vector as a control, and both ALCAM-overexpression constructs. **(C)** Western-blot analysis evidenced that ALCAM-Cherry was predominantly expressed in the cell membrane in Hec1A-ETV5 cells. **(D)** Images of the 3D spheroid-spreading model are illustrated at different time points. In the control cells, the spheroids were almost disaggregated after 5 h, whilst the mean time for disaggregation for the ALCAMcytoless cells was around 18 h, and more than 21 h for the full-length ALCAM cells. **(E)** Effect of ALCAM overexpression on migration was assessed by a 3D spheroid-spreading model on 200 μm fibronectin-coated stripes. On the left, box plot of the speed of migration for each cell line. On the right, representative images at time 0 h and at 24 h are illustrated and the spreading reached at 24 h is outlined in the images. Migration decreased in cells transfected with full-length ALCAM (^**^p<0.01) and ALCAMcytoless (^*^p<0.05) compared to control (m/s, meter/second) **(F)** Left: Two Cherry transiently transfected cells were put into contact and allowed to form adhesion. After 4 and 10 min, we applied an increasing measurable force (nN) up to the disruption of the formed cell doublet. Right: Full-length ALCAM-overexpression increased cell-cell adhesion at all points, as shown by quantification of the separation force (^***^p<0.001). However, ALCAMcytoless only increased cell-cell adhesion at 4 min and showed no differences with control line at 10 min. **(G)** Western-blot analysis of ALCAM-overexpression in Hec1A-ETV5 cells demonstrated a significant decrease in the levels of ERK-phosphorylation.

To understand the role of ALCAM at the invasive process, we recovered its expression in the invasive Hec1A-ETV5 model by using two ALCAM-overexpression vectors: one containing a full-length ALCAM (full-ALCAM) and another containing the extracellular and transmembrane regions (ALCAMcytoless), both cloned in pmCherry-N1 vectors (Figure 4A). When transiently transfected in Hec1A-ETV5 cells, ALCAM-overexpression was localized predominantly in the plasma membrane (Figure 4B-4C). To evaluate the effects of ALCAM-recovery in ETV5-overexpressing cells, we used 3D *in vitro* approaches that closely mimic the *in vivo* settings. We used a spheroid model to quantitatively study the spreading of cell aggregates on fibronectin-coated stripes. In addition to reproduce characteristics of the *in vivo* environment, this model allows us to analyze the competition between cell–cell and cell–substratum adhesion on tissue spreading [38]. In both conditions (ALCAM full-length and ALCAMcytoless) the speed was significantly decreased compared to the control cells (Figure 4D-4E). However, the larger difference was found in the full-length ALCAM-transfected cells. In fact, we observed that the mean time of disaggregation of the Cherry-control spheroids was around 5 h, while ALCAM full-length and ALCAMcytoless expressing aggregates needed approximately 21 h and 18 h, respectively (Figure 4D). Moreover, the pattern of spreading in control cells presented a more spindle-shaped phenotype, with larger presence of individual cells and protrusions, highly dynamic transient cell-cell contacts, migration in a sheet fashion and presentation of chemotactic abilities. In contrast, full-ALCAM and ALCAMcytoless cells presented an increased intercellular adhesion phenotype and a more collective pattern of spreading (Figure 4D, Supplementary Videos 1-3). Consequently, ALCAM recovery clearly impaired the migratory abilities of ETV5-overexpressing cells.

To verify these effects we evaluated how ALCAM-rescue affects the formation and strength of cell-cell adhesion. We used two micropipettes to put into contact two isolated cells maintained in suspension, to avoid cell-matrix interactions, and initiate adhesion. Single cells were chosen under a fluorescence microscope to verify the Cherry-transfection. The mean separation force (SF) is used as a read-out of the strength of adhesion for a precise contact time. In all the cases, the SF required to disrupt the cell doublet increased with the contact time. We observed that at 4 min both ALCAM-transfected cells presented significantly larger adhesion than the control line (Figure 4F). Cells expressing full-length ALCAM displayed stronger intercellular adhesion, as shown by the two-fold increase in their SF compared to control cells, and were more adhesive than cells expressing ALCAMcytoless. In contrast, at 10 min only the full-length ALCAM-transfected cells presented significantly stronger adhesion than the control. Thus, we showed that ALCAM shedding was important to reduce the adhesive properties of the cells. In this particular context, the cytoplasmic tail plays an important role in cell-cell adhesion formation or maturation.

The molecular pathway by which ETV5 promotes its invasive functions in EC has been recently postulated by Alonso-Alconada et al. [39]. The authors described how ETV5 works through the BDNF–TrkB–ERK1/2 axis by inducing ERK1/2 phosphorylation, leading to the promotion of migration and invasion. In this work, we observed that ALCAM-overexpression was concomitant with a decrease in p-ERK, without reverting to an epithelial phenotype (Figure 4G), but impairing the migratory and increasing the adhesive properties promoted by ETV5. Although overexpression of the ALCAMcytoless is sufficient to reduce the levels of p-ERK and to decrease spheroid spreading, ALCAM full-length is necessary to increase cell-cell adhesion with time.

## DISCUSSION

The principal components of adherens junctions in epithelial cells are E-cadherin and β-catenin. E-cadherin is a calcium-dependent transmembrane receptor that interacts homotypically to the E-cadherin of adjacent cells and heterotypically to cytoplasmic-binding proteins, including β-catenin, to transduce external signals within the cell and provide a physical link to the cytoskeleton [40]. Loss of epithelial polarity and decreased E-cadherin lead to EMT, they have been described to promote invasion and are related to poor prognosis and high-grade EC tumors [41]. In addition, the upregulation of the ETV5 Ets transcription factor has been linked to EMT and associated with myometrial infiltration in EC [42]. Our previous *in vitro* and *in vivo* studies evidenced that ETV5 promotes EMT by modulation of ZEB1 expression and consequent E-cadherin repression, leading to a complete reorganization of cell-cell and cell-substrate contacts and in the acquisition of migratory and invasive capabilities [33, 34]. Moreover, ETV5 has been shown to co-localize with MMP-2 and -9 at the invasive front of the tumor [42]. The high expression of both metalloproteinase and COX-2, another direct target of ETV5, has been correlated to poor prognosis and relapse in EC [43, 44].

A critical role has been evidenced in the process of EMT and E-cadherin expression downregulation, by SNAIL-related zinc-finger transcription factors (SNAIL and SLUG). The overexpression of SNAIL and SLUG has been seen closely associated with the reduced expression of E-cadherin in EC, and particularly at the invasive front of the tumor [45, 46]. Specifically, SNAIL nuclear immunoexpression has been described to take place concomitantly with a decrease in E-cadherin expression at the invasive front of stage IB EEC [45].

In this study, we used an extracellular-ALCAM antibody to assess the non-cleaved protein expression by immunohistochemistry in two different tumor areas from 116 EEC primary tumors, i.e. at the superficial and the invasive front. Then, its expression was related to a set of epithelial (E-cadherin, β-catenin) and mesenchymal (ETV5, COX-2, MMP-2, MMP-9, SNAIL, and SLUG) EEC relevant markers. While at the superficial tumor, uncleaved ALCAM maintained a correlation with the major adhesion complex E-Cadherin/β-catenin; at the invasive tumor, where a more mesenchymal microenvironment takes place, it correlated with COX-2, SNAIL, MMP-9 and ETV5 mesenchymal markers. Interestingly, in the moderately-poorly differentiated tumors E-cadherin and ALCAM were positively correlated at the superficial area, whilst ETV5 and MMP-9 presented a significant negative correlation with extracellular ALCAM at the invasive front. Moreover, we evidenced that the correlation between ALCAM and MMP-9 was only significant in poor prognostic scenarios, i.e. in moderately-poorly differentiated tumors, in patients presenting myometrial invasion >50%, and ETV5-positive tumors. Furthermore, at the invasive front, we demonstrated that ALCAM-negativity was an independent prognostic marker of myometrial invasion together with the tumor grade.

ALCAM-negativity was directly associated to an increased shedding of the protein, as demonstrated by the increased expression of sALCAM in uterine aspirates from patients with moderately-poorly differentiated EEC presenting myometrial invasion >50%. In our study, ROC analysis corroborated sALCAM as a significant predictor of myometrial invasion (AUC=0.80). These findings are particularly interesting as uterine aspirates are the preferable biopsy used in EC diagnosis and the assessment of sALCAM might open the avenue to develop a potential tool to determine pre-operatively the myometrial infiltration (sensitivity 87.0%, specificity 70.6%) at the time of diagnosis, even in earlier EC stages [47]. In addition, sALCAM and MMP-9 were significantly strongly correlated in the same patients presenting myometrial invasion >50%. Actually, sALCAM has been detected in FFPE [22] and in several biofluids [31, 48, 49], and related to tumor progression and poor patient outcome. Disruption of ALCAM–ALCAM interactions between cells promotes tumor cell motility and metastasis, then shedding may predict tumor progression at a molecular level. In fact, ectopic expression of mutant ΔN-ALCAM in melanoma cells induced loss of cellular aggregation, increased motility in skin reconstructs and lung metastasis formation at the expense of primary tumor [50].

*In vivo* and *in vitro* ETV5-overexpressing models of EEC were selected. Only in the ETV5-overexpressing invading tumors, uncleaved ALCAM was decreased at the invasive front, concomitant with an increase in MMP-9 expression. MMP-9 expression has been associated with increased metastatic potential in many cancer types [51]. Its activity modulates cell-cell and cell-matrix attachment and has been described to regulate the cell invasion and migration processes. Here, we could hypothesize that MMP-9 is responsible of ALCAM shedding, in invasive scenarios.

Moreover, we proved that ALCAM-recovery in ETV5-overexpressing cells decreased migration, increased cell-cell adhesion, and presented a more collective pattern of spreading. In addition, we evidenced that a larger adhesion force was necessary to separate the full-length ALCAM-transfected cell doublet, indicating its capacity to promote intercellular adhesion strengthening and manifesting the crucial role of its cytoplasmic tail in the maturations of cell-cell adhesion. In fact, the cytoplasmic tail of ALCAM has been described to interact with the actin cytoskeleton, and could be essential in translation of external mechanical forces into the cell to activate biochemical signals [50, 52].

These differences in cell-cell adhesion, in the cell speed and the pattern of migration could be partially explained by the decrease of the active levels of p-ERK. The link between the role of BDNF in the modulation of EMT induced by ETV5 and the acquisition of cell migratory/invasive capabilities through p-ERK phosphorylation has been established [39]. The known role of the BDNF–TrkB–ERK1/2 axis on neuronal plasticity and organogenesis might be mediating pro-metastatic events in endometrial cancer and as consequence the decrease in the p-ERK levels would lead to a less aggressive phenotype [39]. Moreover, some authors evidenced that sustained MAPK activation could lead to enhanced induction of proteolytic enzymes in the surrounding environment in tumors and specifically, the inhibition of ERK activation by MEK inhibitor PD098059 has been described to partially block MMP-9 production and attenuate the *in vivo* invasiveness of head and neck squamous cancer cells [53].

Although further research is needed to define the exact mechanisms, *in vitro* and *in vivo* models evidenced that ALCAM participates in cell-cell adhesion and collective cell migration, and suggested that to allow invasion in the frontier between tissues-restricted carcinoma and disseminated tumor cells, a dynamic and adaptive switch between ALCAM expression at the cell surface and cleaved ALCAM by MMP-9 might take place. In fact, cancer cell invasion is currently viewed as a heterogeneous and adaptive process [54] in which, the plasticity in cell adhesion, the dynamics of the cytoskeleton and the mechanotransduction of external stimuli are key processes. Taking into account that myometrial invasion is highly associated with poor prognosis and to a limited therapeutic response, our research opens an avenue for the use of ALCAM-recovery as a therapeutic approach to control tumor invasion.

## MATERIALS AND METHODS

### Human endometrial cancer samples

Formalin-fixed paraffin-embedded tissue samples from 116 EEC patients were recruited from the Hospital del Mar (Barcelona, Spain). For each patient, we selected 4 macroscopically differentiated areas: 2 superficial, which were obtained from the core of tumor; and 2 invasive, which were obtained from the tumor in contact with the invasive front.

Forty uterine aspirates, from EEC patients were collected prior to surgery by aspiration in the Vall Hebron University Hospital (Barcelona, Spain), with a Cornier Pipelle (Eurogine Ref. 03040200). PBS1x was added (1:1) and centrifuged 20 min at 2,500 rcf to separate the soluble fraction.

All samples were obtained after the approval of the ethical committee of each institution. The clinicopathologic characteristics of all patients are detailed in Supplementary Table 4.

### Immunohistochemical staining

Tissue microarray and immunohistochemistry were performed as previously described [34]. ALCAM, E-cadherin, β-catenin, ETV5, COX-2, MMP-2, MMP-9, SLUG, and SNAIL immunohistochemical expressions were analyzed in both areas of the tumor (superficial and invasive). Immunohistochemical staining for ALCAM was conducted with MOG/07 antibody (ab49496; Abcam, Cambridge, MA, USA) directed against the extracellular domain of the protein. Specifically, the antibody is raised against a 200-amino-acid-long peptide from the extracellular region of ALCAM, detecting the full-ALCAM or non-cleaved protein. Antigen retrieval and primary antibody incubation were performed as previously described [32]. To detect MMP-2, MMP-9, and ETV5, citrate buffer pH 9 was used for antigen retrieval. Then the sections were incubated with 1:100 rAb ETV5 (H-100; Santa Cruz Biotechnology, CA, USA), 1:50 mAb MMP-2 (CA-4001; Abcam, Cambridge, MA, USA), and 1:50 rAb MMP-9 (3852; Cell Signaling Technology, Beverly, MA, USA) for 1h or 2h (ETV5) at room temperature. Detection of COX-2, SLUG, and SNAIL was performed with antigen retrieval citrate pH 6 buffer. The sections were incubated overnight using 1:100 gAb COX-2 (M-19; Santa Cruz Biotechnology, CA, USA), 1:250 rAb SLUG (ab27568; Abcam), 1:125 gAb SNAIL (ab53519; Abcam) for 1h at room temperature. Primary antibodies mAb E-cadherin and mAb β-Catenin (36 and 14; Roche, Basel, Switzerland) were used with Ventana Benchmark automated slide stainer, 24 min at 36°C.

A pathologist evaluated the expression of each protein using immunoreactive scores (IRS). ALCAM, E-Cadherin, SNAIL, SLUG, and COX-2 expressions were evaluated from the product of the staining intensity (ranging from 1-3) and the percentage [0-100%] of neoplastic cells with positive staining. The product of the two assessments yielded final values on a scale ranging from [0–300]. ETV5, β-catenin, MMP-2 and MMP-9 were only evaluated based on the percentage, in scale IRS [0-100%]. For the selected candidates (ALCAM, ETV5 and MMP-9), the IRS was dichotomized (positive/negative), by using the average of their staining (obtained from the superficial and the invasive staining) and the cut-off values were established with the first quartile of its distribution, in each case. The cut-off for each protein was: ALCAM positive ≥ 65, ETV5 positive ≥ 25, MMP-9 positive >5.

### Soluble ALCAM detection in uterine aspirates

The ALCAM Duoset ELISA kit (R&D Systems, Minneapolis, USA) was used to detect ALCAM levels in the supernatant of the uterine aspirates according to manufacturers' instructions. All samples were diluted 1:100 in BSA 1%.

### Zymography

Proteins with metalloproteinase activity were identified by gelatin zymography and equal quantities of protein, from the uterine aspirates, were size fractionated as previously described [35].

### Cell lines, constructs and transient cell lines generation

The human Hec1A and the Hec1A-ETV5 EEC cells were generated and maintained as described in Monge et al. [35]. To monitor non-invasively tumor grafts of Hec1A and Hec1A-ETV5 cell lines, cells were infected with lentiviruses bearing pLenti CMV V5-LUC Blast (w567-1) (Addgene, Cambridge, MA, USA) to constitutively express the luciferase reporter gene, as previously described [55]. For transient ALCAM-overexpression in Hec1A-ETV5, we amplified ALCAM full-length and ALCAM extracellular-transmembrane domains, without the cytoplasmic tail (ALCAMcytoless), from an EEC tissue, using the forward primer 5′-GCAACTCGAGATGGAATCCAAGGGGGCCA-3′, and 2 reverse primers 5′-GCTTGAATTCCGGCTTCAGTTTTGTGATTGTT-3′ and 5′- GCTTGAATTCGCAGCCAGTAGACGACACCAG-3′, respectively. Both amplified regions were inserted in pmCherry-N1 vector (Clontech Laboratories, Mountain View, CA, USA) with *XhoI* and *EcorI*. Transfection was performed using Lipofectamine 2000 (Invitrogen) following manufacturers' instructions.

### Orthotopic murine model and bioluminescent imaging

All the procedures were performed according to the guidelines of the Spanish Council for Animal Care and the institutional guidelines for animal welfare (CEEA 23/16). A total of 9 six-week-old female athymic nude mice (Charles River Laboratories, Inc, Wilmington, MA) were inoculated by transmyometrial injection of Hec1A luciferase control cells (n=5) and stably transfected Hec1A-ETV5 luciferase cells (n=4) as described in [56]. Mice were sacrificed 3 weeks after the injection. All extracted tissues were formalin-fixed, stained with haematoxylin and eosin, and evaluated by a pathologist. After cells injection and before sacrifice, a weekly follow-up was carried out using the IVIS system (Xenogen Corporation) coupled to Living Imaging software 4.2 (Xenogen Corporation) in order to detect tumor growth by bioluminescent imaging. Luciferin (Firefly Luciferin, Caliper Lifescience Corp, Hopkinton, MA, USA) was used as the substrate for the luciferase expressing tumor cells and injected intraperitoneally at a concentration of 150 mg/kg in PBS.

### Western-blot and protein cell extraction

Cell extraction and western-blot were performed as previously described [33]. Incubation with primary antibodies was performed at 4°C overnight using 1:500 mAb ALCAM (MOG/07, Abcam, Cambridge, MA, USA); 1:2000 rAb a-Tubulin, 1:2000 rAb ERK1/2, 1:1000 rAb p-ERK1/2 (Cell Signaling Technology, Beverly, MA, USA), 1:2500 rAb Lamin (Santa Cruz, CA, USA), 1:5000 rAb mCherry ([57]; an aliquot of the antibody was kindly provided by Drs. Anna Santamaría and Erich A. Nigg). Nuclear, membrane and cytosolic fractionation of transfected Hec1A-ETV5 cells was performed by using a Subcellular Fractionation Kit (Pierce) as per the manufacturer's instructions.

### Measurement of the separation force between cells

Forces were measured on the stage of an inverted epifluorescence microscope (Leica) equipped with objectives of 63x (PL FLUOTAR; NA/0.7; C PLAN NA/0.75) and with a cooled CCD C5985 (Hamamatsu) or Coolpix 5000 camera (Nikon) as previously described [58]. To preserve intact the cell surface proteins, cells were dissociated with Cell dissociation enzyme-free buffer (Gibco, NY, USA) and then transferred in working medium (CO2-independent medium, Invitrogen, CA, USA) and used immediately. Two isolated cells were put into contact and allow forming adhesion for specific period times. Aspiration was monitored continuously by a pressure sensor (model DP103-38; Validyne) during the separation process and the values recorded for each of the last two cycles in the series (*P*n-1 and *P*n) were used to calculate the separation force (SF) for each doublet using the equation: SF = *π* (*d*/2)2 (*P*n-1+*P*n)/2 where *d* is the inside diameter of the left pipette. Results for 15–30 measurements were used to obtain the mean SF for a specific contact time.

### Aggregate spreading

Aggregates were obtained from 5 mL of cell suspension in CO2-equilibrated culture medium at a concentration of 4 × 10^5^ cells per milliliters in 25 mL Erlenmeyer flasks and placed in a gyratory orbital shaker at 75 rpm at 37°C for 22 h. The flasks were pretreated with 2% dimethylchlorosilane in chloroform to prevent adhesion of cells to the glass surface.

For the preparation of coated glass substrates: twenty-five millimeter circular glass coverslips were sonicated in ethanol for 5 min, dried at ambient temperature, and exposed to deep UV for 5 min. Fibronectin (Sigma-Aldrich, Missouri, USA) coating was performed using a 0.1 mg/mL solution of fibronectin in PBS solution (pH 7.4) for 1 h. Mixed coating of fibronectin and PEG-PLL (PLL(20)-g[3.5]-PEG(2), Surface Solution) was done by mixing at various rates a 0.1 mg/mL fibronectin in phosphate buffer solution (PBS; pH 7.4) and a 0.1 mg/mL PEG-PLL in Hepes solution (pH 7.3) for 1 h. Coverslips were then rinsed with PBS (pH 7.4).

As previously described [59], aggregates were deposited randomly on striped fibronectin-coated coverslips in a magnetic imaging chamber (Chamlide CMB, CM-B25-1) filled with CO2-equilibrated culture medium. Spreading was observed using a NIKON confocal microscope with an x10 air objective and a 37°C heating cube system. Bright field images were recorded for 36 h with a CCD camera (Luca-R, Andor) using NIS-Elements software. The increased spreading was measured at different time points with Image J software (National Institutes of Health, Bethesda), which allowed calculating the speed of migration for each cell line.

### Immunofluorescence

After 48 h of transfection, cells seeded onto glass coverslips were fixed with 4% paraformaldehyde, blocked in PBS-BSA 4% and incubated with DAPI for 15 min at RT in dark. Coverslips were mounted using the Aqua/Poly Mount medium (Polysciences Europe GmbH, Eppelheim, Germany). Fluorescence images were captured with Spectral Confocal Microscope FV1000 (Olympus, Hamburg, Germany).

### Statistical analysis

For continuous variables: univariate and multivariate linear regression analyses were performed to evaluate the association between ALCAM and epithelial or mesenchymal markers at the superficial and the invasive front. For each cohort, a stepwise method was used to select the explanatory variables based on analysis of variance, checking the normality (Kolmogorov-Smirnov) and the independence of residuals (Durbin-Watson).

Receiver operating characteristic (ROC) curve analysis was conducted on the individual soluble ALCAM (sALCAM) expression detected in uterine aspirates at diagnosis, to evaluate the potential of this parameter as a marker to discriminate patients presenting myometrial infiltration. To explore its relationship with the different forms of MMP-9 present in the uterine aspirates, we performed a factor analysis with extraction of principal component (PCA) and then, a regression analyses with sALCAM levels. The normal distribution of the data was verified (Kolmogorov-Smirnov) and levels were ln-transformed for linear analyses. Non-parametric test (Mann Whitney) were used for comparisons of data sets whose distributions deviate from normal. Measurements were made in triplicates in three independent experiments and presented with the Mean±SD (except for the separation force experiments, which were presented by Mean±SEM).

For categorical variables: the relationships between ALCAM expression at the superficial and the invasive front and clinicopathological parameters were tested in univariate analysis by Chisquare and Fisher's exact test. Stratified analysis was performed to study the molecular features ALCAM/MMP-9/ETV5 and the crosstalk between ALCAM/MMP-9 and clinicopathological parameters. The odds ratios (OR), with their confidence interval (IC 95%) were obtained through bootstrapping methods. Logistic regression multivariate analysis was done for the clinicopathological parameters and the biomarkers status to determine independent predictors for myometrial infiltration.

All statistical analyses were performed using the IBM SPSS Statistics 21. All two-sided p-values < 0.05 were considered statistically significant.

## SUPPLEMENTARY MATERIALS FIGURE, VIDEOS AND TABLES









## Abbreviations

EC: endometrial cancer
EEC: endometrioid endometrial cancer
SF: separation force
sALCAM: soluble ALCAM
ALCAMcytoless: ALCAM without the cytoplasmic tail

## References

[1] Siegel RL, Miller KD, Jemal A (2016). Cancer statistics, 2016. CA Cancer J Clin.

[2] Abal M, Llauradó M, Doll A, Monge M, Colas E, González M, Rigau M, Alazzouzi H, Demajo S, Castellví J, García A, Ramón y Cajal S, Xercavins J (2007). Molecular determinants of invasion in endometrial cancer. Clin Transl Oncol.

[3] Colas E, Pedrola N, Devis L, Ertekin T, Campoy I, Martínez E, Llauradó M, Rigau M, Olivan M, Garcia M, Cabrera S, Gil-Moreno A, Xercavins J (2012). The EMT signaling pathways in endometrial carcinoma. Clin Transl Oncol.

[4] Hanahan D, Weinberg RA (2011). Hallmarks of cancer: The next generation. Cell.

[5] Francí C, Takkunen M, Dave N, Alameda F, Gómez S, Rodríguez R, Escrivà M, Montserrat-Sentís B, Baró T, Garrido M, Bonilla F, Virtanen I, García de Herreros A (2006). Expression of SNAIL protein in tumor–stroma interface. Oncogene.

[6] Christofori G (2006). New signals from the invasive front. Nature.

[7] Bowen MA, Bajorath J, D'Egidio M, Whitney GS, Palmer D, Kobarg J, Starling GC, Siadak AW, Aruffo A (1997). Characterization of mouse ALCAM (CD166): The CD6-binding domain is conserved in different homologs and mediates cross-species binding. Eur J Immunol.

[8] Swart GW (2002). Activated leukocyte cell adhesion molecule (CD166/ALCAM): Developmental and mechanistic aspects of cell clustering and cell migration. Eur J Cell Biol.

[9] Skonier JE, Bowen MA, Emswiler J, Aruffo A, Bajorath J (1996). Recognition of diverse proteins by members of the immunoglobulin superfamily: Delineation of the receptor binding site in the human CD6 ligand ALCAM. Biochemistry.

[10] van Kempen LC, Nelissen JM, Degen WG, Torensma R, Weidle UH, Bloemers HP, Figdor CG, Swart GW (2001). Molecular Basis for the Homophilic Activated Leukocyte Cell Adhesion Molecule (ALCAM)-ALCAM Interaction. J Biol Chem.

[11] Zimmerman AW, Nelissen JM, van Emst-de Vries SE, Willems PH, de Lange F, Collard JG, van Leeuwen FN, Figdor CG (2004). Cytoskeletal restraints regulate homotypic ALCAM-mediated adhesion through PKCalpha independently of Rho-like GTPases. J Cell Sci.

[12] Te Riet J, Zimmerman AW, Cambi A, Joosten B, Speller S, Torensma R, van Leeuwen FN, Figdor CG, de Lange F (2007). Distinct kinetic and mechanical properties govern ALCAM-mediated interactions as shown by single-molecule force spectroscopy. J Cell Sci.

[13] Nelissen JM, Peters IM, de Grooth BG, van Kooyk Y, Figdor CG (2000). Dynamic regulation of activated leukocyte cell adhesion molecule-mediated homotypic cell adhesion through the actin cytoskeleton. Mol Biol Cell.

[14] Tudor C, te Riet J, Eich C, Harkes R, Smisdom N, Bouhuijzen Wen ger J, Ameloot M, Holt M, Kanger JS, Figdor CG, Cambi A, Subramaniam V (2014). Syntenin-1 and ezrin proteins link activated leukocyte cell adhesion molecule to the actin cytoskeleton. J Biol Chem.

[15] Te Riet J, Helenius J, Strohmeyer N, Cambi A, Figdor CG, Müller DJ (2014). Dynamic coupling of ALCAM to the actin cortex strengthens cell adhesion to CD6. J Cell Sci.

[16] Thelen K, Georg T, Bertuch S, Zelina P, Pollerberg GE (2008). Ubiquitination and Endocytosis of Cell Adhesion Molecule DM-GRASP Regulate Its Cell Surface Presence and Affect Its Role for Axon Navigation. J Biol Chem.

[17] Piazza T, Cha E, Bongarzone I, Canevari S, Bolognesi A, Polito L, Bargellesi A, Sassi F, Ferrini S, Fabbi M (2005). Internalization and recycling of ALCAM/CD166 detected by a fully human single-chain recombinant antibody. J Cell Sci.

[18] Rosso O, Piazza T, Bongarzone I, Rossello A, Mezzanzanica D, Canevari S, Orengo AM, Puppo A, Ferrini S, Fabbi M (2007). The ALCAM shedding by the metalloprotease ADAM17/TACE is involved in motility of ovarian carcinoma cells. Mol Cancer Res.

[19] Weidle UH, Eggle D, Klostermann S, Swart GW (2010). ALCAM/CD166: Cancer-related issues. Cancer Genomics and Proteomics.

[20] van Kempen LC, van den Oord JJ, van Muijen GN, Weidle UH, Bloemers HP, Swart GW (2000). Activated leukocyte cell adhesion molecule/CD166, a marker of tumor progression in primary malignant melanoma of the skin. Am J Pathol.

[21] Kristiansen G, Pilarsky C, Wissmann C, Stephan C, Weissbach L, Loy V, Loening S, Dietel M, Rosenthal A (2003). ALCAM/CD166 is up-regulated in low-grade prostate cancer and progressively lost in high-grade lesions. Prostate.

[22] Hansen AG, Freeman TJ, Arnold SA, Starchenko A, Jones-Paris CR, Gilger MA, Washington MK, Fan KH, Shyr Y, Beauchamp RD, Zijlstra A (2013). Elevated ALCAM shedding in colorectal cancer correlates with poor patient outcome. Cancer Res.

[23] King JA, Ofori-Acquah SF, Stevens T, Al-Mehdi AB, Fodstad O, Jiang WG (2004). Activated leukocyte cell adhesion molecule in breast cancer: Prognostic indicator. Breast Cancer Res.

[24] Carbotti G, Orengo AM, Mezzanzanica D, Bagnoli M, Brizzolara A, Emionite L, Puppo A, Centurioni MG, Bruzzone M, Marroni P, Rossello A, Canevari S, Ferrini S (2013). Activated leukocyte cell adhesion molecule soluble form: A potential biomarker of epithelial ovarian cancer is increased in type II tumors. Int J Cancer.

[25] van den Brand M, Takes RP, Blokpoel-deRuyter M, Slootweg PJ, van Kempen LCL (2010). Activated leukocyte cell adhesion molecule expression predicts lymph node metastasis in oral squamous cell carcinoma. Oral Oncol.

[26] Kahlert C, Weber H, Mogler C, Bergmann F, Schirmacher P, Kenngott HG, Matterne U, Mollberg N, Rahbari NN, Hinz U, Koch M, Aigner M, Weitz J (2009). Increased expression of ALCAM/CD166 in pancreatic cancer is an independent prognostic marker for poor survival and early tumour relapse. Br J Cancer.

[27] Tachezy M, Effenberger K, Zander H, Minner S, Gebauer F, Vashist YK, Sauter G, Pantel K, Izbicki JR, Bockhorn M (2012). ALCAM (CD166) expression and serum levels are markers for poor survival of esophageal cancer patients. Int J Cancer.

[28] Ofori-Acquah SF, King JA (2008). Activated leukocyte cell adhesion molecule: A new paradox in cancer. Transl Res.

[29] Kulasingam V, Zheng Y, Soosaipillai A, Leon AE, Gion M, Diamandis EP (2009). Activated leukocyte cell adhesion molecule: A novel biomarker for breast cancer. Int J Cancer.

[30] Chaker S, Kashat L, Voisin S, Kaur J, Kak I, MacMillan C, Ozcelik H, Siu KW, Ralhan R, Walfish PG (2013). Secretome proteins as candidate biomarkers for aggressive thyroid carcinomas. Proteomics.

[31] Tachezy M, Zander H, Marx AH, Stahl PR, Gebauer F, Izbicki JR, Bockhorn M (2012). ALCAM (CD166) expression and serum levels in pancreatic cancer. PLoS One.

[32] Devis L, Moiola CP, Masia N, Martinez-Garcia E, Santacana M, Stirbat TV, Brochard-Wyart F, García Á, Alameda F, Cabrera S, Palacios J, Moreno-Bueno G, Abal M (2017). Activated leukocyte cell adhesion molecule (ALCAM) is a marker of recurrence and promotes cell migration, invasion and metastasis in early stage endometrioid endometrial cancer. J Pathol.

[33] Colas E, Muinelo-Romay L, Alonso-Alconada L, Llaurado M, Monge M, Barbazan J, Gonzalez M, Schoumacher M, Pedrola N, Ertekin T, Devis L, Ruiz A, Castellvi J (2012). ETV5 cooperates with LPP as a sensor of extracellular signals and promotes EMT in endometrial carcinomas. Oncogene.

[34] Pedrola N, Devis L, Llauradó M, Campoy I, Martinez-Garcia E, Garcia M, Muinelo-Romay L, Alonso-Alconada L, Abal M, Alameda F, Mancebo G, Carreras R, Castellví J (2015). Nidogen 1 and Nuclear Protein 1: Novel targets of ETV5 transcription factor involved in endometrial cancer invasion. Clin Exp Metastasis.

[35] Monge M, Colas E, Doll A, Gonzalez M, Gil-Moreno A, Planaguma J, Quiles M, Arbos MA, Garcia A, Castellvi J, Llaurado M, Rigau M, Alazzouzi H (2007). ERM/ETV5 up-regulation plays a role during myometrial infiltration through matrix metalloproteinase-2 activation in endometrial cancer. Cancer Res.

[36] Parkes C, Kamal A, Valentijn AJ, Alnafakh R, Gross SR, Barraclough R, Moss D, Kirwan J, Hapangama DK (2018). Assessing Estrogen-Induced Proliferative Response in an Endometrial Cancer Cell Line Using a Universally Applicable Methodological Guide. Int J Gynecol Cancer.

[37] Muinelo-Romay L, Colas E, Barbazan J, Alonso-Alconada L, Alonso-Nocelo M, Bouso M, Curiel T, Cueva J, Anido U, Forteza J, Gil-Moreno A, Reventos J, Lopez-Lopez R (2011). High-risk endometrial carcinoma profiling identifies TGF-β1 as a key factor in the initiation of tumor invasion. Mol Cancer Ther.

[38] Ryan PL, Foty RA, Kohn J, Steinberg MS (2001). Tissue spreading on implantable substrates is a competitive outcome of cell-cell vs. cell-substratum adhesivity. Proc Natl Acad Sci.

[39] Alonso-Alconada L, Eritja N, Muinelo-Romay L, Barbazan J, Lopez-Lopez R, Matias-Guiu X, Gil-Moreno A, Dolcet X, Abal M (2014). ETV5 transcription program links BDNF and promotion of EMT at invasive front of endometrial carcinomas. Carcinogenesis.

[40] Théard D, Raspe MA, Kalicharan D, Hoekstra D, van IJzendoorn SC (2008). Formation of E-cadherin/beta-catenin-based adherens junctions in hepatocytes requires serine-10 in p27(Kip1). Mol Biol Cell.

[41] Sugihara T (2016). Loss of Adherens Junction Protein E-Cadherin is a Biomarker of High-Grade Histology and Poor Prognosis in Endometrial Cancer. Endometrial Cancer Ann Clin Lab Res.

[42] Planagumà J, Liljeström M, Alameda F, Bützow R, Virtanen I, Reventós J, Hukkanen M (2011). Matrix metalloproteinase-2 and matrix metalloproteinase-9 codistribute with transcription factors RUNX1/AML1 and ETV5/ERM at the invasive front of endometrial and ovarian carcinoma. Hum Pathol.

[43] Aglund K, Rauvala M, Puistola U, Ångström T, Turpeenniemi-Hujanen T, Zackrisson B, Stendahl U (2004). Gelatinases A and B (MMP-2 and MMP-9) in endometrial cancer—MMP-9 correlates to the grade and the stage. Gynecol Oncol.

[44] Karahan N, Güney M, Baspinar S, Oral B, Kapucuoglu N, Mungan T (2007). Expression of gelatinase (MMP-2 and MMP-9) and cyclooxygenase-2 (COX-2) in endometrial carcinoma. Eur J Gynaecol Oncol.

[45] Montserrat N, Mozos A, Llobet D, Dolcet X, Pons C, de Herreros AG, Matias-Guiu X, Prat J (2012). Epithelial to mesenchymal transition in early stage endometrioid endometrial carcinoma. Hum Pathol.

[46] Tanaka Y, Terai Y, Kawaguchi H, Fujiwara S, Yoo S, Tsunetoh S, Takai M, Kanemura M, Tanabe A, Ohmichi M (2013). Prognostic impact of EMT (epithelial-mesenchymal-transition)-related protein expression in endometrial cancer. Cancer Biol Ther.

[47] Martinez-Garcia E, Lesur A, Devis L, Campos AR, Cabrera S, Van Oostrum J, Matias-Guiu X, Gil-Moreno A, Reventos J, Colas E, Domon B (2016). Development of a sequential workflow based on LC-PRM for the verification of endometrial cancer protein biomarkers in uterine aspirate samples. Oncotarget.

[48] Arnold Egloff SA, Du L, Loomans HA, Starchenko A, Su PF, Ketova T, Knoll PB, Wang J, Haddad AQ, Fadare O, Cates JM, Lotan Y, Shyr Y (2016). Shed urinary ALCAM is an independent prognostic biomarker of three-year overall survival after cystectomy in patients with bladder cancer. Oncotarget.

[49] Witzel I, Schröder C, Müller V, Zander H, Tachezy M, Ihnen M, Jänicke F, Milde-Langosch K (2012). Detection of activated leukocyte cell adhesion molecule in the serum of breast cancer patients and implications for prognosis. Oncology.

[50] van Kempen LC, Meier F, Egeblad M, Kersten-Niessen MJ, Garbe C, Weidle UH, Van Muijen GN, Herlyn M, Bloemers HP, Swart GW (2004). Truncation of activated leukocyte cell adhesion molecule: A gateway to melanoma metastasis. J Invest Dermatol.

[51] Moulik S, Pal S, Biswas J, Chatterjee A (2014). Role of ERK in Modulating MMP 2 and MMP 9 with Respect to Tumour Invasiveness in Human Cancer Cell Line MCF-7 and MDA-MB-231. J Tumor.

[52] Gardel ML, Schneider IC, Aratyn-Schaus Y, Waterman CM (2010). Mechanical integration of actin and adhesion dynamics in cell migration. Annu Rev Cell Dev Biol.

[53] Yao J, Xiong S, Klos K, Nguyen N, Grijalva R, Li P, Yu D (2001). Multiple signaling pathways involved in activation of matrix metalloproteinase-9 (MMP-9) by heregulin-β1 in human breast cancer cells. Oncogene.

[54] Friedl P, Alexander S (2011). Cancer Invasion and the Microenvironment: Plasticity and Reciprocity. Cell.

[55] Alonso-Alconada L, Muinelo-Romay L, Madissoo K, Diaz-Lopez A, Krakstad C, Trovik J, Wik E, Hapangama D, Coenegrachts L, Cano A, Gil-Moreno A, Chiva L, Cueva J (2014). Molecular profiling of circulating tumor cells links plasticity to the metastatic process in endometrial cancer. Mol Cancer.

[56] Cabrera S, Llaurado M, Castellvi J, Fernandez Y, Alameda F, Colas E, Ruiz A, Doll A, Schwartz S, Carreras R, Xercavins J, Abal M, Gil-Moreno A (2012). Generation and characterization of orthotopic murine models for endometrial cancer. Clin Exp Metastasis.

[57] Hübner NC, Wang LH, Kaulich M, Descombes P, Poser I, Nigg EA (2010). Re-examination of siRNA specificity questions role of PICH and Tao1 in the spindle checkpoint and identifies Mad2 as a sensitive target for small RNAs. Chromosoma.

[58] Chu YS, Thomas WA, Eder O, Pincet F, Perez E, Thiery JP, Dufour S (2004). Force measurements in E-cadherin-mediated cell doublets reveal rapid adhesion strengthened by actin cytoskeleton remodeling through Rac and Cdc42. J Cell Biol.

[59] Douezan S, Guevorkian K, Naouar R, Dufour S, Cuvelier D, Brochard-Wyart F (2011). Spreading dynamics and wetting transition of cellular aggregates. Proc Natl Acad Sci U S A.

