# Supplementary material for: ALCAM shedding at the invasive front of the tumor is a marker of myometrial infiltration and promotes invasion in endometrioid endometrial cancer
Source: Oncotarget. 2018 Mar 30;9(24):16648–64. doi: 10.18632/oncotarget.24625 (PMC5908276; doi:10.18632/oncotarget.24625)
Supplement: Supplementary file 1 [file oncotarget-09-16648-s001.pdf]

ALCAM shedding at the invasive front of the tumor is a marker of myometrial infiltration and promotes invasion in endometrioid endometrial cancer

SUPPLEMENTARY MATERIALS

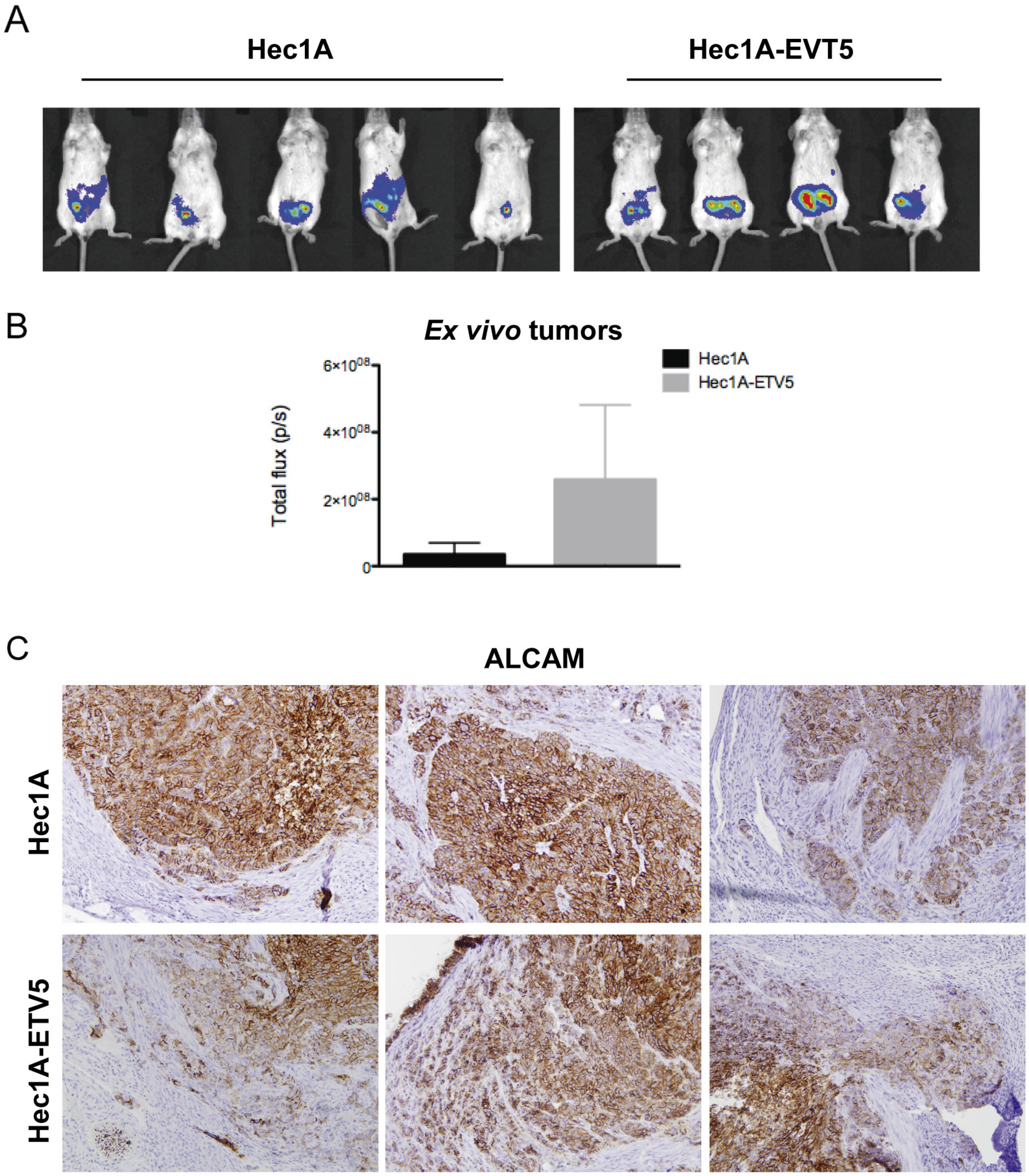

Supplementary Figure 1: Hec1A and Hec1A-ETV5 orthotopic murine model. (A) IVIS images of the injected mice evidenced a larger tumor growth in the ETV5-overexpressing cells. **(B)** Quantification of the luminescence of the primary tumors ex vivo evidenced a higher intensity in the ETV5-overexpressing mice (p=0.064). **(C)** Upper: Images of ALCAM expression in Hec1A control mice, presenting less invasion and scattered clusters of cells. Down: Images of ALCAM in Hec1A-ETV5 mice, with higher rate of invasion and disseminated cells, with a finger-strand pattern. ALCAM staining was more homogeneous in the control model with lower invasion and more collective invasion.

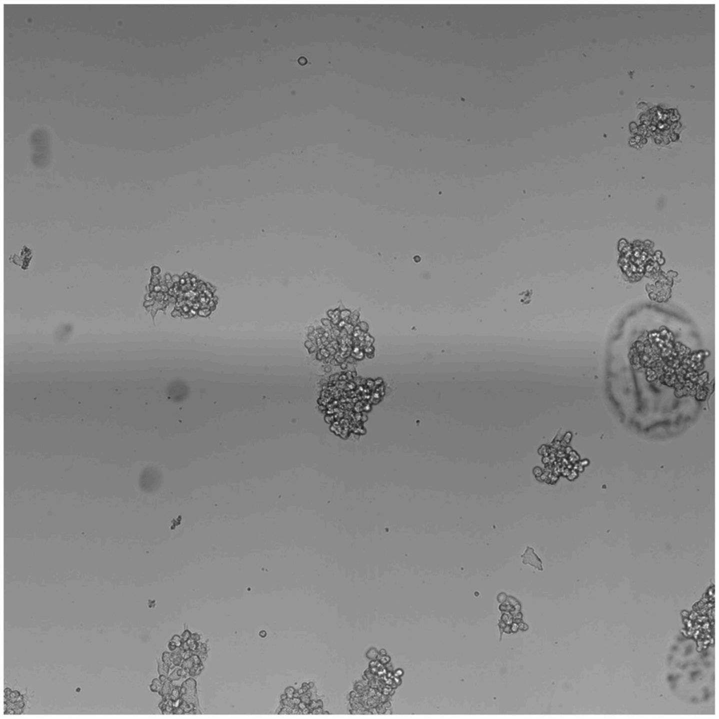

Supplementary Video 1: Hec1A-ETV5 ALCAMcytoless-Cherry spreading aggregate on fibronectin-coated striped followed for 36 h.

See Supplementary Video 1

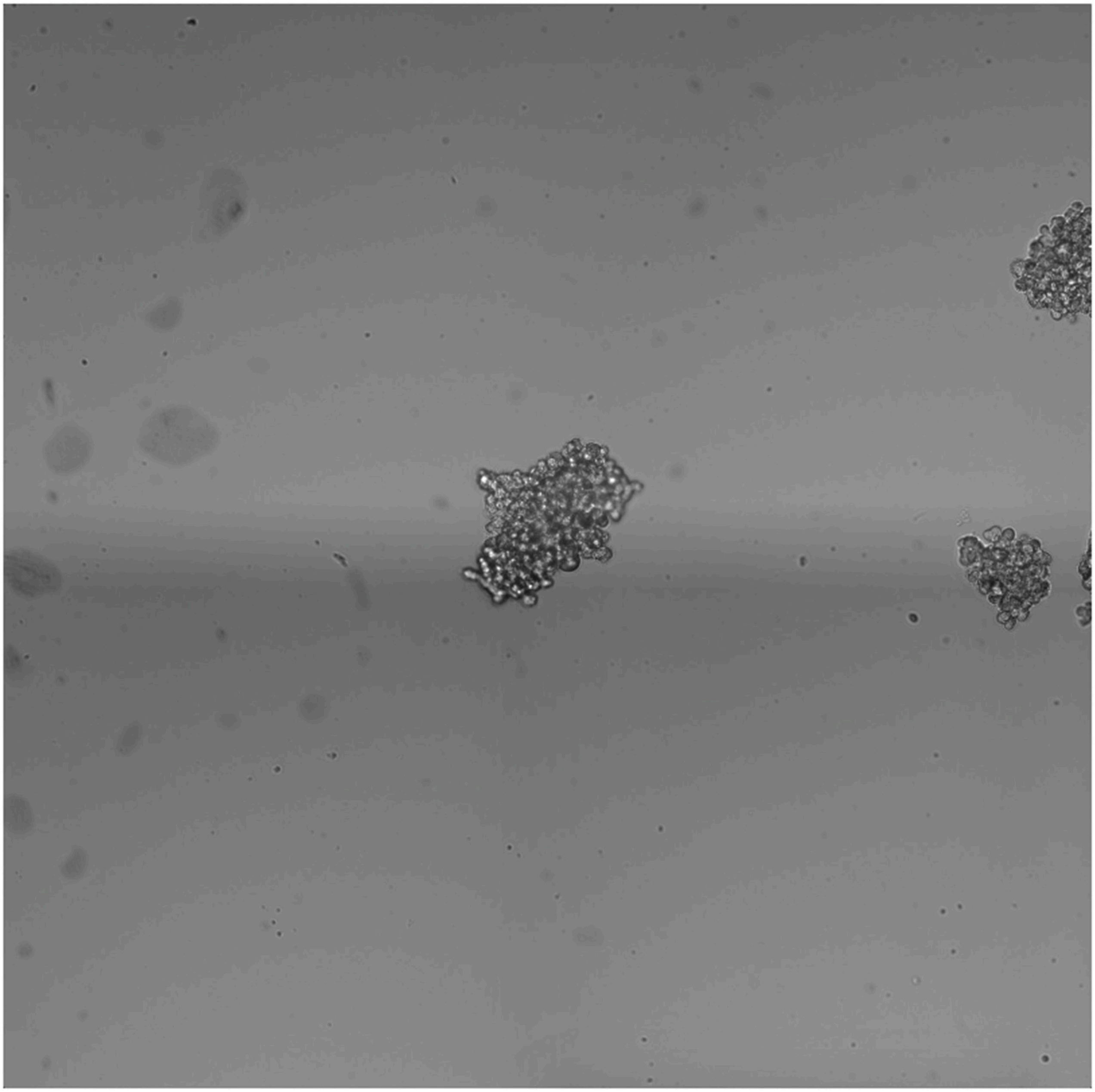

Supplementary Video 2: Hec1A-ETV5 ALCAM-Cherry spreading aggregate on fibronectin-coated striped followed for 36 h.

See Supplementary Video 2

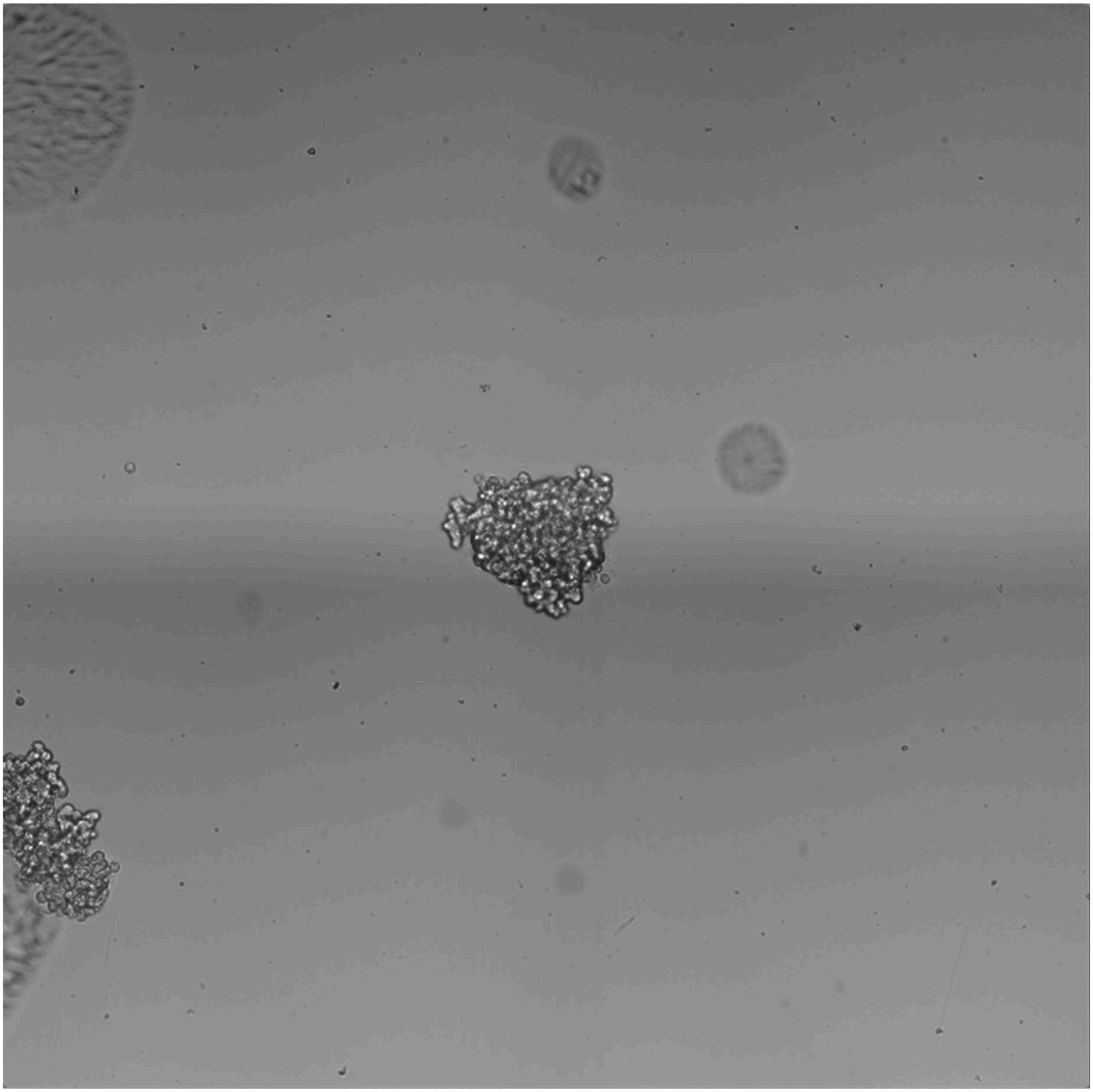

Supplementary Video 3: Hec1A-ETV5 NCherry spreading aggregate on fibronectin-coated stripes followed for 36 h.

See Supplementary Video 3

Supplementary Table 1: Patterns of ALCAM in the tumor: univariate linear regression

	All cases		Grade I		Grade > I	
	**β**	**p-value**	**β**	**p-value**	**β**	**p-value**
**Superficial area of the tumor**						
β-catenin	0.419	< 0.001***	0.394	< 0.001***	0.410	0.013*
E-cadherin	0.346	< 0.001***	0.275	0.014*	0.462	0.004**
ETV5	0.026	0.791	0.033	0.780	0.027	0.878
COX-2	0.109	0.248	0.153	0.180	0.013	0.938
MMP-9	-0.176	0.060	-0.014	0.906	-0.395	0.015*
MMP-2	0.247	0.008**	0.251	0.026*	0.246	0.143
SNAIL	0.214	0.028*	0.192	0.112	0.272	0.109
SLUG	0.067	0.472	0.061	0.595	0.320	0.054
**Invasive front of the tumor**						
β-catenin	0.144	0.174	0.181	0.157	0.017	0.933
E-cadherin	0.160	0.125	0.173	0.182	0.111	0.546
ETV5	-0.034	0.748	0.118	0.365	-0.408	0.025*
COX-2	0.210	0.038*	0.221	0.073	0.188	0.310
MMP-9	-0.242	0.018*	-0.137	0.280	-0.438	0.014*
MMP-2	0.035	0.723	0.029	0.808	-	-
SNAIL	0.300	0.005*	0.409	0.001**	0.005	0.980
SLUG	0.036	0.711	0.018	0.883	0.168	0.343

Supplementary Table 2: Patterns of ALCAM in the superficial and invasive areas of the tumor: multivariate linear regression

**Superficial area of the tumor**							
	**Summary model**			**Coefficients**			
	R	R^2 adj.	p-value	B	CI95%	β	p-value
**All cases**	0.437	0.183	<0.001***				
Predictors							
β-catenin				1.765	(1.045; 2.484)	0.437	< 0.001***
SNAIL						0.170	0.088
E-cadherin						0.166	0.096
MMP-2						0.133	0.183
**Grade**							
**Well-differentiated**	0.394	0.144	<0.001***				
Predictors							
β-catenin				1.592	(0.733; 2.451)	0.394	<0.001***
E-cadherin						0.089	0.449
MMP-2						0.131	0.264
**Moderately-poorly differentiated**	0.445	0.174	0.007**				
Predictors							
E-cadherin				0.673	(0.201; 1.146)	0.445	0.007**
β-catenin						0.276	0.109
MMP-9						-0.276	0.108
**Invasive front of the tumor**							
	**Summary model**			**Coefficients**			
	R	R^2 adj.	p-value	B	CI95%	β	p-value
**All cases**	0.458	0.179	<0.001***				
Predictors							
SNAIL				0.396	(0.075; 0.717)	0.259	0.016*
MMP-9				-1.330	(-2.415; -0.244)	-0.250	0.017*
COX2				0.522	(0.089; 0.955)	0.253	0.019*
**Grade**							
**Well-differentiated**	0.409	0.152	0.001**				
Predictors							
SNAIL				0.573	(0.231; 0.915)	0.409	0.001**
**Moderately-poorly differentiated**	0.559	0.259	0.008**				
Predictors							
ETV5				-1.175	(-2.283; -0.067)	-0.363	0.039*
MMP-9				-1.987	(-3.917; -0.057)	-0.353	0.044*

Supplementary Table 3: MMP-9 and ALCAM in uterine aspirates

Matrix correlation MMP-9 forms (*)

	**MMP-9 D**	**MMP-9 NGAL**	**MMP-9 L**	**MMP-9 A**
**MMP-9 D**		0.815***	0.826***	0.552***
**MMP-9 NGAL**	0.815***		0.699***	0.545***
**MMP-9 L**	0.826***	0.699***		0.543***
**MMP-9 A**	0.552***	0.545***	0.543***	

(*) ln-transformed

ALCAM correlation with MMP-9

	**Univariate linear regression**					
	**All cases**		**Myometrial infiltration <50%**		**Myometrial infiltration >50%**	
	**R**	**p-value**	**R**	**p-value**	**R**	**p-value**
MMP-9 Dimeric	0.355	0.025*	0.298	0.246	0.484	0.019*
MMP-9 NGAL	0.407	0.009**	0.313	0.221	0.446	0.033*
MMP-9 Latent	0.385	0.014*	0.282	0.272	0.326	0.129
MMP-9 Active	0.100	0.541	0.197	0.449	0.095	0.666
C1 Regression Factor Score	0.447	0.004**	0.322	0.207	0.575	0.004**

Supplementary Table 4: Human endometrial cancer samples

TMA Description	
**Variable**	**Σ (%)**
**FIGO Stage**	116 (100.0)
Stage IA	56 (48.3)
Stage IB	41 (35.3)
Stage II	15 (12.9)
Stage III	4 (3.4)
**Grading**	116 (100.0)
Grade I	79 (68.1)
Grade II	24 (20.7)
Grade III	13 (11.2)
**Myometrial Infiltration**	116 (100.0)
No	58 (50.0)
Yes	58 (50.0)
**Uterine aspirates**	
**Variable**	**Σ (%)**
**FIGO Stage**	40 (100.0)
Stage IA	15 (37.5)
Stage IB	12 (30.0)
Stage II	7 (17.5)
Stage III	3 (7.5)
Stage IV	3 (7.5)
**Myometrial Infiltration**	40 (100.0)
No	17 (42.5)
Yes	23 (57.5)
